# Clinical and Demographic Profile of Volkmann’s Ischemic Contractures Presenting at National Institute of Rehabilitation Medicine, Islamabad, Pakistan

**DOI:** 10.29252/wjps.9.2.166

**Published:** 2020-05

**Authors:** Muhammad Saaiq

**Affiliations:** Department of Plastic Surgery, National Institute of Rehabilitation Medicine (NIRM), Islamabad, Pakistan

**Keywords:** Volkmann’s ischemic contracture, Acute compartment syndrome, Flexor pronator slide, Tendon-transfers

## Abstract

**BACKGROUND:**

Established Volkmann’s ischemic contractures (VICs) represent the eventuality of neglected acute compartment syndrome (ACS) of the forearm. This study assessed the clinical and demographic presentation of VICs.

**METHODS:**

This study was conducted at Department of Plastic Surgery, National Institute of Rehabilitation Medicine, Islamabad, Pakistan over a period of three years and included all patients of either gender who presented with VICs and analyzed various corrective procedures instituted as surgical remedies.

**RESULTS:**

Among 37 included patients, 83.78% were male and 16.21% were female (mean age: 16.51±9.1 years). The underlying causes of the initial traumatic insults were tight bandages employed by traditional bone setters for treating forearm fractures (83.78%), high voltage electric burns involving hands/forearms (13.51%) and supracondylar fracture with vascular compromise (2.70%). Majority of patients belonged to Holden type 2 (97.29%) and Tsuge type 2 VICs (48.68%). The most common sufferers of VICs were young, illiterate males coming from rural regions. Treatment for forearm fractures by traditional bonesetters constituted the most common underlying cause. Most of the patients were managed with combination of procedures including tendon transfers, excision of the fibrosed muscles, tenolysis and neurolysis of median and ulnar nerves. Tendon transfers were the commonest corrective procedures instituted.

**CONCLUSION:**

This study highlighted the gravity of this largely preventable but neglected catastrophe and there is a need to institute robust preventive measures to address the issue. Emphasis should be on creation of public awareness and robust education of health care providers to ensure correct primary management of upper limb trauma.

## INTRODUCTION

Established Volkmann’s ischemic contractures (VICs) represent the eventuality of neglected acute compartment syndrome (ACS) of the forearm. Its hallmark is the presence of irreversible tissue necrosis, which is manifested by a spectrum of muscle contractures and myoneural impairments of variable proportions. The associated nerve dysfunction can result either from the initial ischemic insult or the subsequent fibrotic encasements around the nerves. The muscle fibrosis also results in a variety of secondary joint deformities.^1^^,^^2^

Anatomically, the forearm represents an unyielding osteomyofascial compartment that can accommodate a limited volume and withstands a particular level of pressure. Factors such as tight constrictive bandages or casts that exert continued external pressure, local thermal insults, burn eschars, severe forearm edema, reperfusion injury and bleeding from within, all have a net effect of volume expansion or pressure escalation within the unyielding confines of this closed compartment. As a consequence, an acute ACS develops which exerts its ischemic ramifications on the contained muscles, nerves and overlying skin.^2^^-^^5^

The tolerance level of various tissues to ischemic insult varies in terms of duration of critical ischemia. For instance, muscle ischemia produces irreversible damage within 6-12 hours, whereas the nerve ischemia beyond 12 hours would produce irreversible nerve damage. The skin typically develops irreversible changes after 12 hours of severe ischemia.^2^^-^^5^ The current study was undertaken to determine the clinical and demographic presentation of VICs and document the various corrective procedures instituted for managing the contractures and also to generate evidence-base to better understand and address this largely preventable disaster.

## MATERIALS AND METHODS

This descriptive case series study was conducted From October 01, 2016 to September 30, 2019 at the Department of Plastic Surgery, National Institute of Rehabilitation Medicine, Islamabad, Pakistan over a period of three years. It included all patients of either gender, who presented with established VICs. The exclusion criteria were patients with ACS and VIC in evolution. Informed consent was taken from the patients. The study followed the ethical protocols as per Helsinki’s Declaration-2013 revision. The anonymity of participants was guaranteed.

All patients underwent standard clinical evaluation by history, examination and ancillary investigations. Detailed history about important demographic and social parameters was acquired. The variables of interest included age, gender, residential locale (rural, suburban or urban areas), educational status (primary, secondary, literate, illiterate) and the type of initial traumatic insult. All patients were hospitalized for their definitive surgical management. 

The surgeries were undertaken under general anesthesia. Standard volar incision was employed that extended from the medial aspect of the distal arm as far as the wrist. Among all patients, excision of the fibrosed/infarcted muscles, neurolysis of median and ulnar nerves and tenolysis of the affected tendons were performed. Other corrective procedures were performed as dictated by the needs of individual requirements of the patients. 

These included Z-lengthening of the tendons of flexor digitorum profundus (FDP), flexor digitorum superficialis (FDS) and flexor pollicis longus (FPL), various tendon transfers including brachioradialis (BR) to FPL, extensor carpi radialis longus (ECRL) to FDP, and FDS to FDP, flexor pronator tendon slide, wrist arthrodesis, shortening osteotomy of radius and ulna and nerve reconstructions with cable sural nerve grafts. In the procedure of flexor pronator slide, the proximal origins of the contracted flexor pronator muscles were released. 

At the outset of the procedure, the ulnar nerve was identified and released, as is done in a standard nerve decompression. Careful dissection was performed to lift the flexor pronator origin off the medial epicondyle. The joint capsule and medial collateral ligament were preserved. Similarly, the origins of flexor carpi ulnaris (FCU), FDS and FDP were carefully separated from the ulna and interosseous membrane. Furthermore, dissection proceeded on the radial side with separation of the origin of FPL. 

All important neurovascular structures were safeguarded through the dissection. Distally, the pronator quadratus was separated from the distal ulna. The application of appropriate postoperative cast immobilization helped to heal the tendons in their new positions. Postoperatively, patients with tendon transfers, tendon lengthening and flexor-pronator slide were immobilized for 4-6 weeks with above-elbow splints. In case of tendon transfers for fingers and thumb flexion, the postoperative immobilization was ensured with a dorsal block slab, keeping the wrist in 30° flexion, metacarpophalangeal joints (MCPJs) in 70° flexion, the interphalangeal joints (IPJs) in neutral, and thumb in abduction and pronation. 

In case of patients with flexor-pronator slide, immobilization was done with volar slab with the wrist extended by 20°, the MCJPs slightly flexed at 15° and the IPJs were left free. The elbow was kept at 90° and forearm in supination. The thumb was kept in extension and radial abduction. The first wound dressing was changed on 5^th^ postoperative day. Stitches were removed on 14^th^ postoperative day. The splint was maintained for 6-weeks. Following discontinuation of the splint, physiotherapy was instituted to ensure range of motion. 

**Fig. 1:**
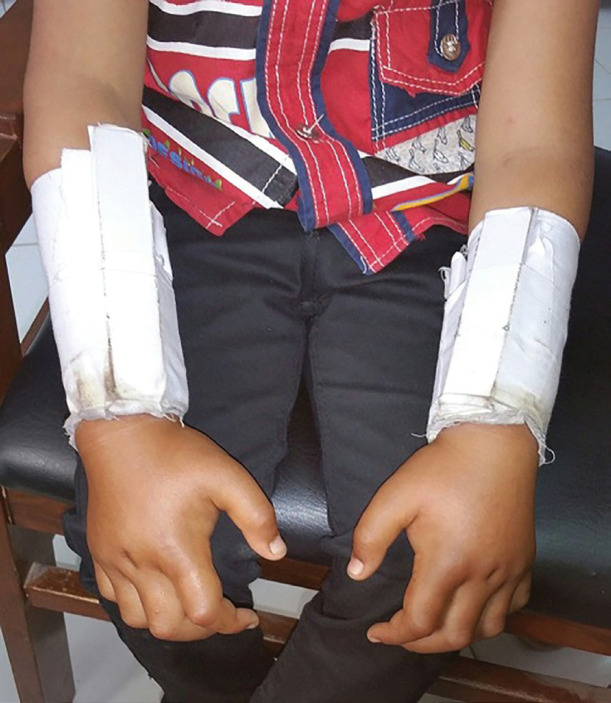
This photograph demonstrates the typical method employed by traditional bone setters. They fix the fractures initially with brutal manipulation to reduce it, followed by putting bamboo sticks around the distal two-thirds of the forearm. These sticks are then surrounded by tight constricting bandages. The proximal one third of the forearm is characteristically spared. Swelling of the hands can be appreciated

The target follow up period was one year. The demographic and clinical profile of the patients and various corrective surgical procedures instituted were all recorded. The data were subjected to statistical analysis using SPSS software (version 11, Chicago, IL, USA). Chi square test was employed to analyse the data and p<0.05 was considered statistically significant (Figures 1). 

## RESULTS 

There were a total of 37 patients with 31 (83.78%) males, and six (16.21%) females. The mean age was 16.51±9.1 years, ranging from 7 to 31 years. Majority of the patients (n=32; 86.48%) belonged to rural communities; whereas, there were five patients from suburban areas (13.51%). None of the patients were reported from urban areas. Regarding educational status of the patients, seven (18.91%) had acquired primary level education, four (10.81%) were literate and 26 (70.27%) were illiterate.

The underlying causes of the initial traumatic insults included application of tight bandages by traditional bone setters for fixing forearm fractures (n=31; 83.78%), high voltage electric burns involving hands/forearms (n=5; 13.51%) and supracondylar fracture with vascular compromise (n=1; 2.70%). The time interval between the onset of VIC and presentation to our hospital ranged from 1 to 7 years with a mean of 2.5 years. There was one case (2.70%) of Holden type 1 VIC, whereas 36 patients (97.29%) belonged to the Holden type 2 category. According to the Tsuge classification system, 13 patients (35.13%) were severe type, 18 patients (48.68%) had moderate type, while six patients (16.21%) suffered from mild VIC. Majority of the patients were managed with a combination of procedures. 

Excision of the fibrosed/infarcted muscle, neurolysis of median and ulnar nerves and tenolysis of the affected tendons were performed among all cases. Other corrective performed procedures included Z-lengthening of various tendons (n=17; 45.94%), BR to FPL tendon transfer (n=27; 72.97%), ECRL to FDP tendon transfer (n=22; 59.45%) and FDS to FDP tendon transfer (n=8; 21.62%), flexor pronator tendon slide (n=5; 13.51%), wrist arthrodesis (n=3; 8%), shortening osteotomy of radius and ulna (n=1; 2.70%) and nerve grafting with sural nerve grafts (n=3; 8%) for restoring protective hand sensation.

## DISCUSSION

This study reflects the high prevalence of VICs in our population. The grave issue continues to plague our population. Our institute is a national referral center for the rehabilitation of disabled individuals coming from across the country. We receive patients from all parts of the country. For instance, the country’s northern regions of Gilgit-Baltistan and Azad Kashmir, the province of Khyber-Pakhtunkhwa, and remote districts of Punjab. 

We also provide services to our primary catchment areas of the twin cities of Islamabad and Rawalpindi. Furthermore, we receive such patients from our neighboring country of Afghanistan. In this study, more frequent involvement of males than females was observed. Our finding conforms to similar observation reported in several published studies from the developing world. For instance, Sharma *et al.*^6^ from India reported a series of nineteen patients with 12 males and 7 females. Similarly Meena *et al.* from Iran reported a series of 12 patients, where 9 were males and 3 were females.^7^

More frequent affliction of males with the devastating condition is probably a reflection of their more frequent involvement in out-door and risky activities that predispose them to the initial inciting traumatic events, which subsequently culminate in the development of VICs.^8^^,^^9^ There was more frequent affliction of the relatively younger segment of the population in this study. Sharma *et al.* from India reported an age-range of 3-25 years, with 18 years as the average age at presentation. Meena *et al.*^7^ from Iran reported 8.3 years as the average age of their patients.^6^

In this study, a significant proportion of patients developed VICs following their treatment for forearm fractures by traditional bone setters in the rural locales. In all these cases, tight constricting bandages had been fashioned with locally available bamboo sticks (Figure 1). The patients gave typical history of severe pain in the hours following application of these circumferential castings; however, they did not seek any immediate medical remedy for the severe alarming pain from any qualified medical professional.

**Fig. 2:**
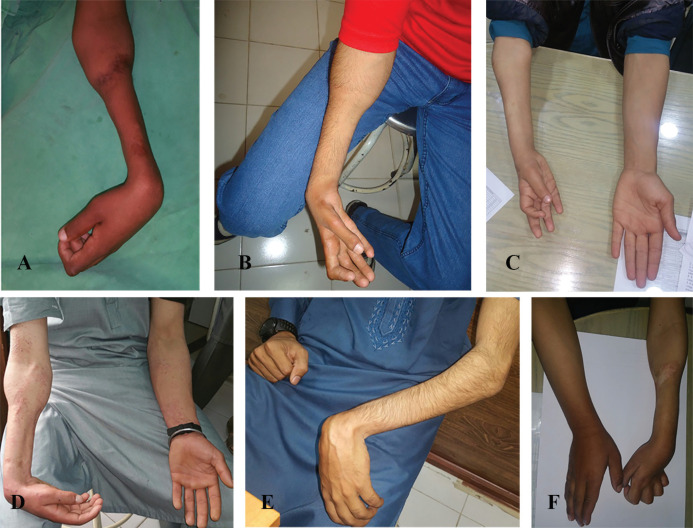
**A:** The left forearm of a 16 years boy with severe VIC showing the characteristic demarcation with normal muscle bulk in the proximal one third and circumferential atrophy involving distal two-thirds of the forearm. There is flexion contracture of the wrist. **B:** The right forearm of a 19 years adolescent male with severe VIC showing the circumferential atrophy of distal two-thirds of forearm. Flexion contractures of the fingers and wrist are visible. **C:** The right forearm of 17 years old boy who had sustained primary ischemic insult seven years ago. Remarkably there is global atrophy and shortening of the forearm and hand as compared to the contralateral normal side. **D:** The right forearm of a 20 years male with VIC of moderate severity. Flexion contracture of the wrist is appreciable. **E:** The left sided VIC in a 25 years old man with fixed flexion contracture of the wrist. **F:** Severe VIC involving the left forearm of 19 years adolescent. There is clawing because of the associated impaired median and ulnar nerves

Among all these cases, the affected tissues directly underlied the area where pressure had been exerted by tight bandages, typically leaving normal proximal area of the unaffected forearm (Figure 2). The current observations conform to similar findings reported by authors from other developing countries.^6^^,^^10^ Sundararaj *et al.*^11^from India and Meena et *al.* from Iran, have reported similar cases of forearm fractures managed by non-doctors as the leading cause of VICs among their patients.^7^

Contrary to the situation in the developing world, among the cases of VICs reported from the developed countries, supracondylar humeral fracture has been identified as the most common cause of VIC. This fracture is relatively common among children aged around 7 years, where the supracondylar area is undergoing active remodeling and is markedly thinner, hence predisposing it to fracture. Approximately 20% of these fractures are associated with some form of vascular compromise.^6^^,^^12^^-^^14^

Electric burns constituted the second most common group of patients with VICs in the current study. We are faced with the menace of burns particularly the high voltage ones, which have many long term repercussions including VICs. Adequate management of such injuries at the very outset helps to avoid such devastating consequences of burn injuries.^9^^,^^15^ In this study VICs were classified by employing a combination of Holden and Tsuge classification systems. 

As the established VICs have a broad range of clinical presentations, no single classification system alone can serve to adequately describe them. Hence combining the Holden and Tsuge systems serves to solve this issue. The Holden classification divides the VICs into two levels of injury. In the level I, the injury is proximal to the zone of ischemia/contracture, as would be typically seen in case of a brachial artery injury. In the level II, the injury is directly onto the zone of ischemia and contracture. In the Tsuge classification, VICs are divided into mild, moderate and severe subtypes based on the extent of muscle necrosis.^1^^-^^4^

The optimal timing of surgical intervention for the VIC patients continues to be debated. In the current study, the shortest time interval between the onset of VIC and presentation to the hospital was one year. Sharma *et al.*^6^ operated majority of their patients within 9 months post-injury, with a range between 1-17 years. Meena *et al.*^7^ operated six patients within 6 months of the initial insult, whereas the remaining five cases were operated after 6 months. Tsuge observed that while deciding the best time for intervention, one has to weight and balance certain key conflicting factors.^4^

For instance, the muscles may sustain even more harm, if we rush for too early surgery; however, in case of nerve involvement, the more severe the affliction the better is earlier intervention. Seddon observed that one should wait for at least 3 months, as spontaneous recovery often begins by that time. However, if there is no recovery, early nerve decompression is required preferably between 3-12 months.^16^ Tendon transfers constituted the work-horse procedure for addressing majority of the moderate contractures.^1^^-^^3^

Mostly, we undertook transfer of extensor tendons to the flexors. When the extensor compartment had not been severely affected with typical sparing of the muscle mass itself, BR and ECRL tendons constituted our first donor choice. They were divided from their distal insertions, transferred to FDP and FPL, respectively. The transfers were secured with Pulvertaft-weaving. FDS to FDP tendon transfers were undertaken, where the FDS muscle was minimally affected and hence usable as a donor for FDP. The ideal candidates for tendon transfers were those who had supple joints of the fingers, thumb and wrist.^1^^-^^3^

In this study, Z-lengthening of the tendons was performed among cases of mild contractures. Each tendon was divided in a well-planned Z-fashion. Subsequently, fingers and thumb were placed in the neutral position and the overlapping tendon lengths were securely sutured together with non-absorbable sutures. Where possible, the Z-lengthening of tendons was performed at different levels for different tendons in such a way that the adjacent repaired tendons did not overlie each other, so as to prevent their tethering together through the course of healing.^1^^-^^3^

The flexor pronator slide operation was performed among five patients. One of these patients had developed Holden type 1 contracture following a supracondylar fracture and four had VICs of Holden type 2. There was good recovery among our patients. Page first described the procedure in 1923.^17^ Sharma *et* *al.* who employed this procedure for moderate VICs with no fixed joint contractures, also observed improvements in dexterity, sensibility, ability to flex and extend the fingers, and grip strength.^6^

They attributed their good outcomes to careful patient selection and adequate postoperative therapy. In this study, shortening osteotomy was performed in one case as an adjunct procedure to fixation of the non-united fractures of radius and ulna. Skeletal shortening of the radius and ulna represents one of the available options to help match the bone length to the shortened fibrotic muscles. One problem with the bone shortening procedures is that the main contracture is within the flexor compartment; however the shortening results in lengthening of both flexor and extensor muscles.^1^^,^^18^

Also, the shortening procedures are not desirable for pediatric age group as the forearm is already shortened by the initial ischemic insult to the bone and its growth plates. The shortening procedures are suitable for long-standing contractures which are not amenable to the soft tissue release procedures.^1^^,^^18^ In this study, wrist arthrodesis was performed in three patients. Proximal row carpectomy was performed as part of the procedure. The published literature also describes some other bone procedures such as the trapeziometacarpal joint fusion, thumb metacarpophalangeal joint fusion and arthrodesis of fingers in functional position. The skeletal procedures are ideally carried out after skeletal maturity.^1^^,^^2^

**Fig. 3:**
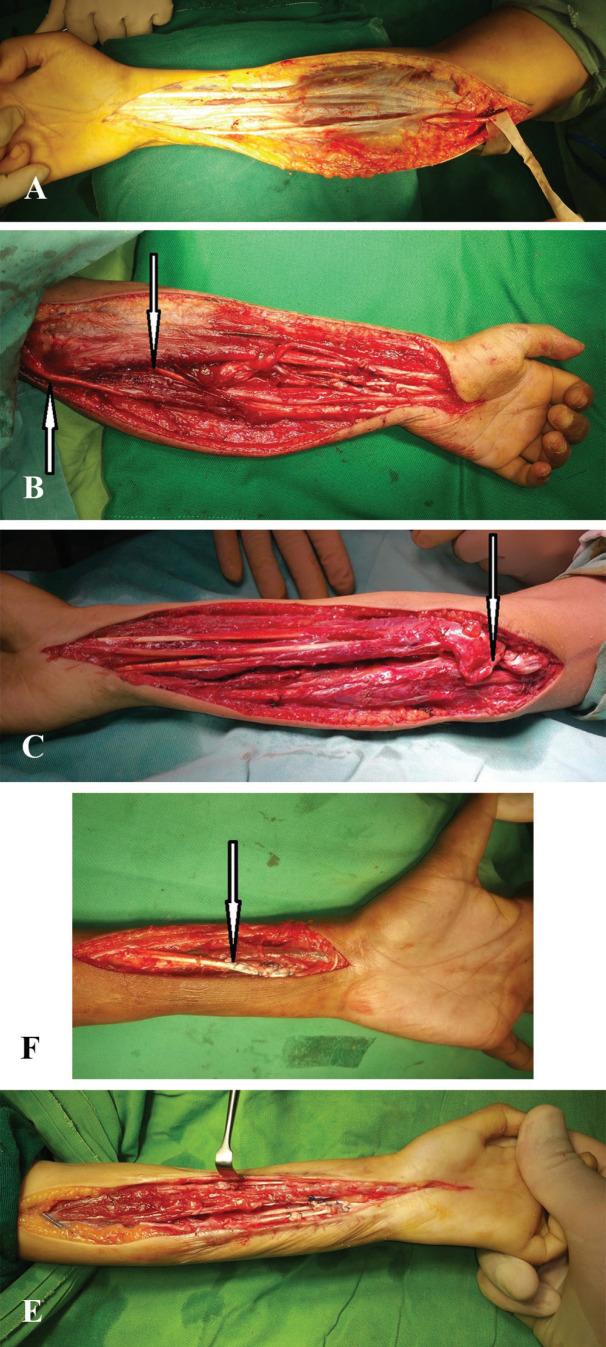
**A: **Characteristic appearance of the plastered tissues seen in the VIC affected forearm. Ulnar nerve at elbow has been isolated.** B:** Tenolysis of tendons and neurolysis of the median and ulnar nerves (arrows point towards the released nerves).** C:** Flexor pronator tendon slide. The arrow points towards the completely released common flexor pronator origin from the medial epicondyle.** D:** Transfer of BR to FPL and ECRL to FDP. The arrow points towards the Pulvertaft-weaving of the transfer.** E:** Z-lengthening of the tendons of FDP, FDS and FPL has been effected

Majority of the patients in the current study showed reasonable improvements in the range of flexion and extension of the fingers, thumb and wrist, manual dexterity, sensory recovery and grip power, following their surgical management (Figure 3). Restoration of completely normal hand function is often not possible, however functional grasp of hand with some protective sensation is usually achievable. The published literature is scare regarding outcome studies on VICs. This is so owing to a host of issues. 

For instance, the limited number of patients with VICs encountered in any single institution, simultaneous and variable presence of both muscle and nerve impairments, need for a combination of procedures in any given patient and lack of possibility of randomization for different treatment protocols. Ultee *et al.* observed that VIC patients who had sufficient remaining muscle, good hand function was restored with procedures that combined infarct excision, tenolysis, neurolysis, and tendon transfer.^19^

Sundararaj *et al.*^11^ similarly observed improvement in sensory function subsequent to neurolysis. “Prevention is better than cure” is a maxim that holds particularly true when we happen to deal with the issue of VICs. Not surprisingly, VICs are almost extinct in clinical practice in the developed world. This is because of their robust preventive strategies based on early detection and prompt treatment of any ACS of the forearm and hand. The issue of VICs however, still continues to plague the developing nations. 

ACS of the forearm and hand needs prompt diagnosis, based on a shrewd clinical acumen of the immediate care providing doctor. Every patient who presents with severe pain of the forearm/hand, which is disproportionate to his primary clinical condition following some traumatic event or iatrogenic procedure, and combined with loss of sensibility should be suspected of having an ACS and hence, managed promptly accordingly. A high index of suspicion for diagnosing ACS and low threshold for performing fasciotomies are the guiding principles.

We showed that the most common sufferers of VICs were young, illiterate males coming from rural backgrounds. Treatment for forearm fractures by traditional bonesetters constituted the most common underlying cause that led to the development of VICs. The most common presentation was with Holden type 2 and Tsuge type 2 contractures. Most of the patients were managed with combination of procedures including tendon transfers, excision of the fibrosed muscles, tenolysis and neurolysis of median and ulnar nerves. This study highlighted the gravity of this largely preventable, but neglected catastrophe. There is a need to institute robust preventive measures to address the issue. Emphasis should be on creation of public awareness and educating the health care providers about the correct primary management of upper limb trauma.

## CONFLICT OF INTEREST

The authors declare no conflict of interest.
